# Immune Checkpoint Inhibitors as Independent and Synergistic Drivers of SJS/TEN: A FAERS-Based Analysis

**DOI:** 10.1101/2025.06.21.25330030

**Published:** 2025-06-30

**Authors:** Eric Milan Mukherjee, Dodie Park, Matthew S. Krantz, Cosby A. Stone, Michelle Martin-Pozo, Elizabeth Phillips

**Affiliations:** 1 Department of Dermatology, Vanderbilt University Medical Center, Nashville, Tennessee, USA; 2 Center for Drug Safety and Immunology, Vanderbilt University Medical Center, Nashville, Tennessee, USA; 3 Department of Medicine, Division of Infectious Diseases, Vanderbilt University Medical Center, Nashville, Tennessee, USA; 4 Institute for Immunology and Infectious Diseases, Murdoch University, Perth, Australia

**Keywords:** Severe Cutaneous Adverse Reactions, Stevens-Johnson Syndrome, Toxic Epidermal Necrolysis, Checkpoint Inhibitors, Pharmacovigilance, FAERS

## Abstract

**Importance:**

Stevens-Johnson Syndrome and Toxic Epidermal Necrolysis (SJS/TEN) are rare, potentially fatal adverse drug reactions. As the use of immune checkpoint inhibitors (ICIs) expands, their role as direct inducers or synergistic contributors to SJS/TEN remains incompletely characterized.

**Objective:**

To determine whether ICIs are independent risk factors for SJS/TEN, evaluate their interactions with known culprit drugs, and assess their impact on latency and mortality.

**Design:**

Cross-sectional analysis of adverse event reports submitted to the U.S. Food and Drug Administration Adverse Event Reporting System (FDA FAERS) between January 2013 and December 2023, sanitized and deduplicated. Logistic regression and Cox models were used to assess predictors of SJS/TEN development, mortality, and latency.

**Setting:**

Global pharmacovigilance reports submitted to FAERS.

**Participants:**

A total of 17,495 unique and de-identified patients reported SJS/TEN, of 13,986,839 total reports.

**Exposures:**

Suspected causative drugs, including ICIs.

**Main Outcomes and Measures:**

Primary outcomes were the adjusted odds of developing SJS/TEN, time-to-event (TTE) of reaction/drug latency, and all-cause mortality. Depending on the analysis, covariates included age, sex, number of concomitant drugs, cancer diagnosis, and specific drug exposures.

**Results:**

Of 17,495 SJS/TEN cases (median age 53 years, 37.6% male), 970 (5.5%) had ICI exposure and 653 (3.7%) listed an ICI as the primary suspect. ICI exposure was associated with developing SJS/TEN (adjusted OR, 6.69; 95% CI, 6.19–7.23) while controlling for age, exposure to strong and weak culprits, number of concomitant drugs, and cancer diagnosis. ICI increases SJS-TEN risk among patients exposed to allopurinol (OR, 4.35; 95% CI, 3.12–6.06) and TMP-SMX (OR, 5.68; 95% CI, 4.05–7.95) with the same covariates. Among patients with small-molecule-induced SJS/TEN, mortality was strongly associated with ICI exposure (particularly exposure to multiple ICI, OR, 7.31; 95% CI, 3.09–17.27). Among all SJS/TEN cases, ICI exposure was associated with delayed onset, compared to cancer patients not exposed to ICI and non-cancer patients (median 20 vs 14 vs 13 days; P < .0001).

**Conclusions and Relevance:**

ICIs are associated with increased SJS/TEN risk, both independently and in combination with known culprit drugs, and may delay disease onset. These findings support increased vigilance in prescribing known culprits alongside ICI.

## Introduction

Immune checkpoint inhibitors (ICIs) are a novel class of antineoplastic drugs that have revolutionized treatment for many late-stage cancers.^1,2^ However, because ICIs deactivate immune tolerance, they are associated with immune-related adverse events (irAE), of which cutaneous irAE are the most common.^3,4^ Some of these reactions imitate Stevens-Johnson syndrome/toxic epidermal necrolysis (SJS/TEN), a rare blistering eruption most often triggered by small-molecule drugs like antibiotics, allopurinol, and antiepileptics.^5,6^ Additionally, ICIs are the fastest-increasing drug class reported in association with SJS/TEN, reflecting both their expanding clinical use and a growing number of serious irAE captured in pharmacovigilance databases.^7^

There is also some controversy over the a distinction between “true” SJS/TEN to ICI and ICI-induced SJS/TEN-like reactions, which may be clinically and pathologically distinct from each other – the latter of which may represent a severe lichenoid or bullous reaction in some cases.^8–12^ Additionally, ICIs may “prime” the risk of cutaneous irAE to known small-molecule culprits, though evidence for this is somewhat scarce. A retrospective study showed antibiotic use in patients receiving ICI increases the risk of some adverse events.^13^ Another study suggested SJS/TEN-like reactions (referred to as progressive immunotherapy-related mucocutaneous eruption or PIRME in that publication) on ICI developed after exposure to a known culprit like allopurinol or trimethoprim-sulfamethoxazole (TMP-SMX), suggesting a two-hit mechanism in which ICIs create a permissive milieu for cutaneous adverse events to known culprit medications.^14,15^

To understand the interaction between ICI and small-molecule drugs in SJS/TEN, we present a comprehensive pharmacovigilance analysis of SJS/TEN using data from the U.S. Food and Drug Administration Adverse Event Reporting System (FAERS) from 2013 to 2023. We assess the role of ICI and known culprit co-exposure on SJS/TEN risk, latency, and mortality. Using multivariable logistic regression, disproportionality analysis, and time-to-event (TTE) analysis, we investigate both the independent and interactive effects of ICI and small-molecule agents in SJS/TEN. Our findings aim to clarify the evolving landscape of drug-induced SJS/TEN and inform future mechanistic, clinical, and regulatory efforts.

## Methods

All coding was done in RStudio 2024.12.0 Build 467 with R version 4.4.2, with GPT4o assistance. Figures were created using the gt (v0.11.1), ggplot2 (v3.5.1) and survminer (v0.5.0) packages, and GPT4o.

### Data

A previously sanitized, deduplicated version of FAERS was used, pared to reports filed between January 2013 and December 2023.^7^

For combination drugs, TMP-SMX was considered the primary suspect if either TMP-SMX was directly coded as the primary suspect, if TMP was coded as the primary suspect and SMX was present on the drug list, or vice versa. For analyses of drug exposure, either TMP or SMX on the drug list was sufficient to consider the patient to be exposed to TMP-SMX. Combination drugs containing phenytoin or allopurinol were also included. Exposure to ICI was coded similarly. ICI exposure by group (PD-1, PD-L1, and CTLA-4) was also noted, and dual ICI exposure was coded by flagging patients exposed to more than one ICI. Biologics were distinguished from small molecules by flagging all drugs that contain a word ending in “mab” or “cept” using regex.

### Cancer Diagnosis

FAERS’ standard_case_indication table was used to flag cases with an associated cancer diagnosis using keywords. After this first pass, we found that 22423 ICI-exposed patients had no cancer diagnosis (11.8% of 189830 ICI-exposed patients). Subsequently, all drugs tied to a known cancer diagnosis were extracted, and the top 1000 by frequency were manually annotated as exclusively used for cancer indications (e.g. checkpoint inhibitors, most kinase inhibitors except JAK inhibitors and those used for idiopathic pulmonary fibrosis) or not (e.g. rituximab, cyclophosphamide, and prednisone, which are commonly used for autoimmune disease). Exact keywords and drugs used can be found in the R markdown file in the online data.

### Co-Exposure Disproportionality Analysis

To evaluate potential interactions between ICI (ipilimumab, nivolumab, pembrolizumab, atezolizumab, cemiplimab, avelumab, durvalumab tremelimumab, dostarlimab, retifanlimab, relatlimab), and drugs known to cause SJS/TEN, we conducted a pairwise co-exposure analysis using reporting odds ratios (ROR). Based on literature, we selected a set of five strong culprits (lamotrigine, TMP-SMX, phenytoin, allopurinol, carbamazepine) and five weak culprits (azithromycin, ciprofloxacin, oxcarbazepine, sulfasalazine, and acyclovir). ICIs were grouped both collectively and by mechanistic subclass (PD-1, PD-L1, CTLA-4, LAG-3, and dual blockade). We conducted two complementary analyses, first evaluating whether the addition of an ICI increased the OR of SJS/TEN in patients on a culprit medication, then evaluating whether the addition of a culprit medication increased the OR in patients receiving an ICI. Two-sided p-values were calculated using Fisher’s exact test and corrected using the Benjamini-Hochberg procedure.

### Logistic Regression

All logistic regressions for the risk of developing SJS/TEN and risk of mortality after developing SJS/TEN were conducted using the glm function in base R with the binomial model. Variance inflation factors (VIFs) for all variables in all models were < 5, suggesting no multicollinearity was detected (eFigure 11). For models with causative drug as a covariate, the top 10 drugs were included, and the rest coded as other (and used as the reference), due to technical limitations.

### Time-to-Event Analysis

After removal of TTE outliers (<1 and >180 days), 4,086 cases had sufficient data for TTE analysis. Cox proportional hazards models were generated using R’s survival package (v3.7–0).

## Results

### Demographics and ICI Exposure

**Figure 1.**
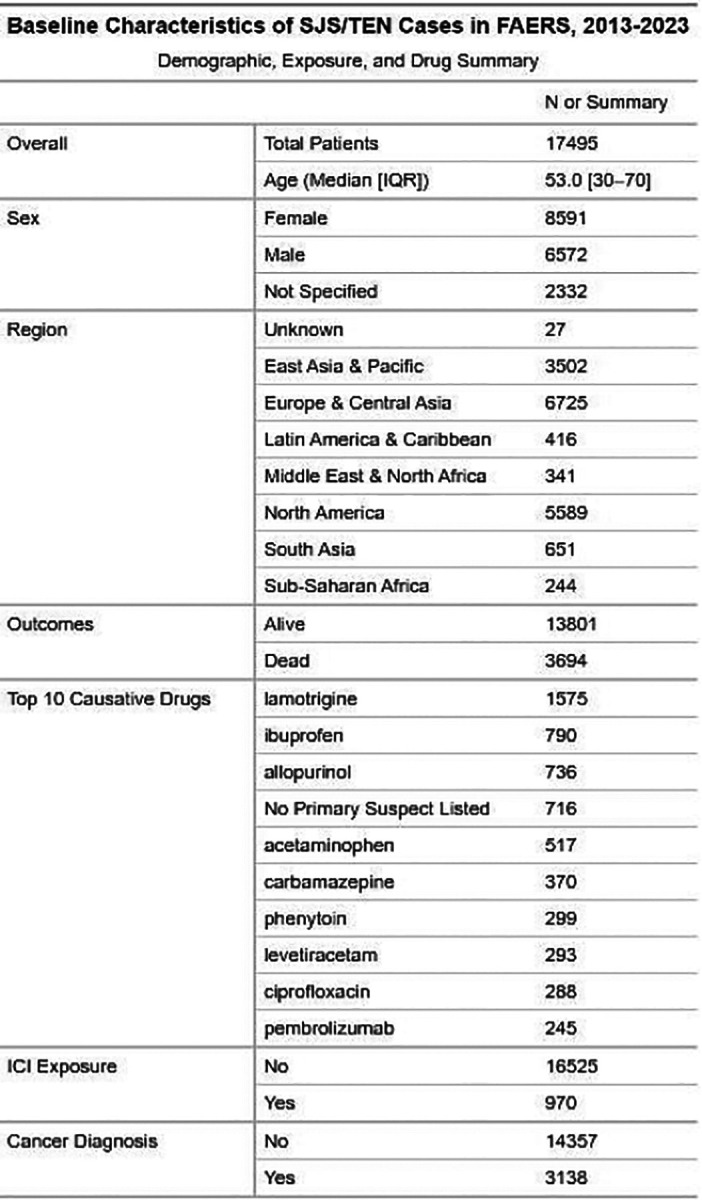

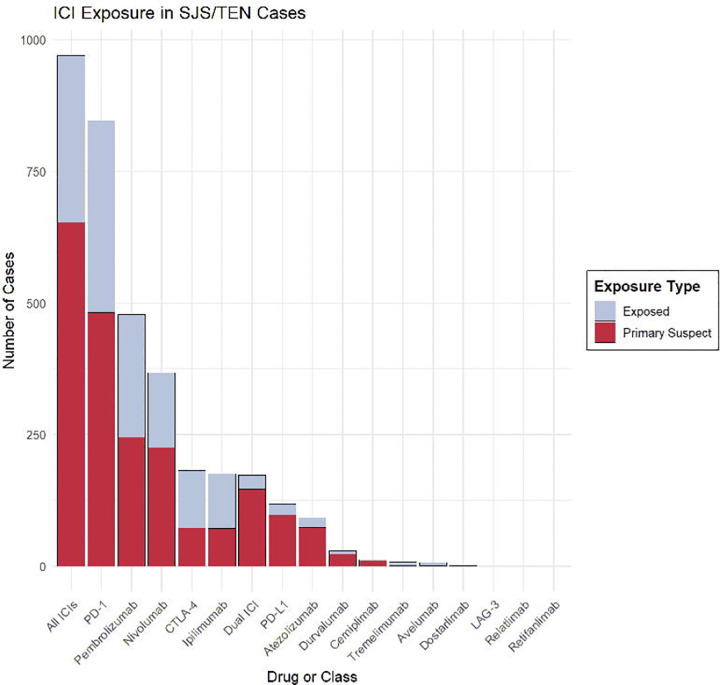
Baseline Characteristics and ICI Involvement SJS/TEN Cases Reported to FAERS (2013–2023). (A) Distribution of ICI-associated SJS/TEN cases by drug and class. Bars indicate the number of cases where a given ICI was reported, with red segments denoting cases in which the ICI is the primary suspect. (B) Demographic, geographic, clinical, and drug exposure characteristics of SJS/TEN cases. IQR = interquartile range, F = female, M = male, NS = not specified.

We identified 17,495 unique SJS/TEN cases reported to FAERS between 2013 and 2023, of a total of 13,986,839 unique reports to FAERS (Figure 1). Median age was 53 years (IQR: 30–70), with slight female predominance (Female: 8591; Male: 6572; Not Specified: 2332). Most reports originated from Europe & Central Asia (6725), North America (5589), and East Asia & Pacific (3502). Mortality was 21.1% overall (3694 deaths). The most common primary suspects were lamotrigine (1545), ibuprofen (790), and allopurinol (736). Of ICI, pembrolizumab made the top 10 with 245 primary suspect cases. ICI exposure was recorded in 970 cases (5.5%), with 653 (3.7%) listing an ICI as the primary suspect. Pembrolizumab (245), nivolumab (225), and ipilimumab (72) were the most frequent primary suspects. By class, PD-1 inhibitors are the most implicated (847 exposed cases, of which 482 are primary suspects), though PD-L1 inhibitors have a higher proportion of implicated cases (118 exposed, 98 primary). Dual ICI regimens also have a high attribution rate (173 exposed, 146 with one of the ICI are coded as the primary suspect). CTLA-4 inhibitors have a lower attribution rate (182 exposed, 73 primary) than other classes.

Supplemental analyses comparing ICI-induced (meaning ICI marked as the primary suspect) and small-molecule-induced SJS/TEN cases revealed demographic distinctions (eFigure 1). ICI-induced cases skewed older and male. ICI-induced cases were more often reported from East Asia and the Pacific than Europe and Central Asia.

### ICI Exposure and the Development of, and Mortality Due to, SJS/TEN

**Figure 2.**
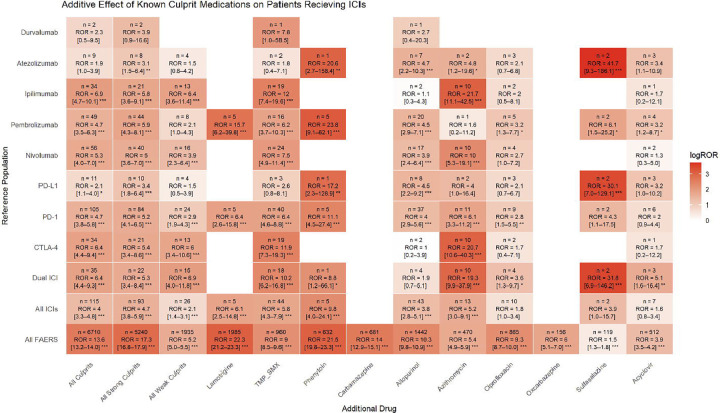

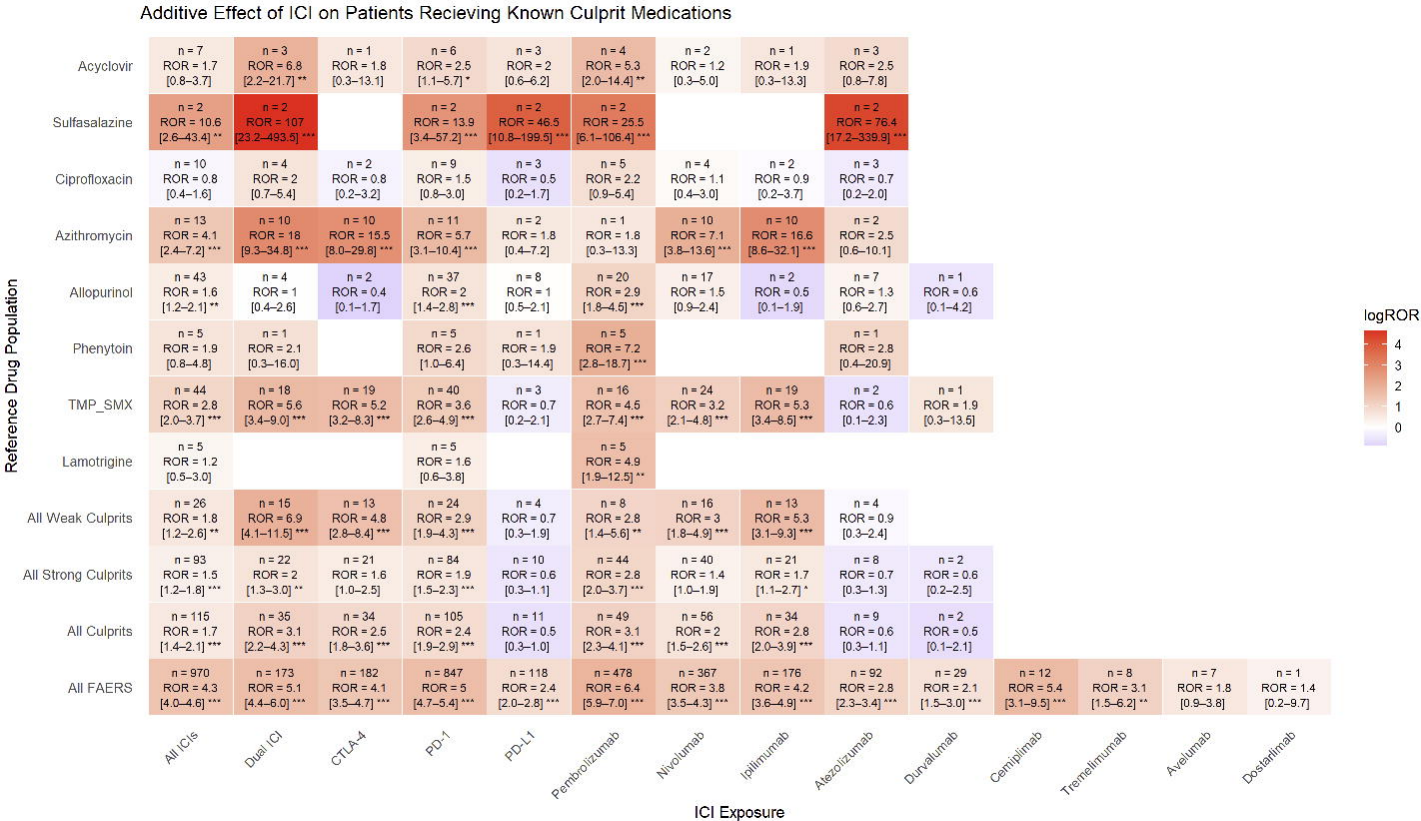
Additive Effects of ICIs and Known Culprit Drugs on Reporting Risk of SJS/TEN. (A) Reporting odds ratios (RORs) for SJS/TEN among patients receiving an ICI (rows) as a reference population, in combination with an additional culprit (columns). (B) RORs for SJS/TEN among patients receiving a culprit (rows), as a reference population, in combination with an additional ICI (columns). In both panels, cells are shaded by the log-transformed ROR, and text shows the case count (n) and estimated ROR with 95% CI. P-values were corrected by the Benjamini-Hochberg procedure within each panel. *p < 0.05; **p < 0.01; ***p < 0.001.

We investigated disproportional reporting of SJS/TEN in patients receiving ICI and/or a set of predefined drugs, divided into strong (TMP-SMX, allopurinol, lamotrigine, phenytoin, and carbamazepine) and weak culprits (azithromycin, ciprofloxacin, oxcarbazepine, sulfasalazine, and acyclovir); this analysis was conducted in both directions (Figure 2). For patients on ICI, exposure to strong (OR = 4.7 [3.8–5.9], P < 0.001) or weak culprits (OR = 2.1 [1.4–3.1], P < 0.01) is disproportionately associated with SJS/TEN. Across patients exposed to any strong culprit, ICI co-exposure resulted in a modest increased odds of SJS-TEN (ROR, 1.5; 95% CI, 1.2–1.8, P < 0.001); patients exposed to a weak culprit had similar odds (ROR = 1.8 [1.2–2.6], P < 0.01). Particularly strong signals with dual ICI exposure were noted with patients on azithromycin (ROR = 18.0 [9.3–34.8], P < 0.001) and sulfasalazine (OR = 107 [23.2–493.5], P < 0.001), though limited by small sample sizes. In both directions, ORs are generally comparable or lower than ORs using the entire FAERS database as the background.

Of these culprits, the ones with the most frequent co-exposure in SJS/TEN patients are allopurinol (n = 43 cases) and TMP-SMX (44). In allopurinol-exposed patients, ICI increased the risk of SJS/TEN fourfold (OR = 4.35 [3.12–6.06]) after adjusting for age, sex, number of concomitant drugs, and cancer diagnosis using multivariate logistic regression (eFigure 2). Similar findings were observed for TMP-SMX (OR = 5.68 [4.05–7.95]) (eFigure 3). In both cases, cancer diagnosis was negatively associated with developing SJS/TEN (OR = 0.37 [0.32–0.43] for allopurinol, OR = 0.39 [0.33–0.46 for TMP-SMX), despite ICI being positively associated.

**Figure 3.**
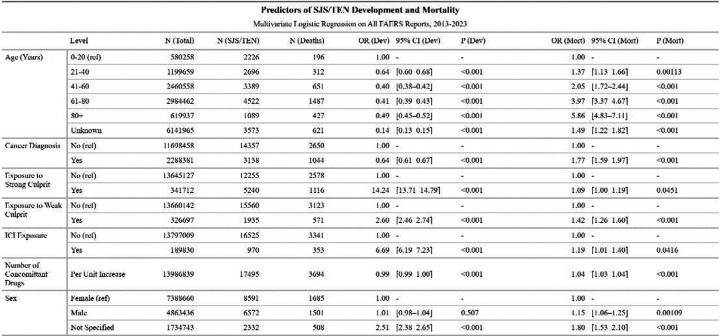
Predictors of SJS/TEN Development and Mortality. Multivariable logistic regression was used to assess factors associated with SJS/TEN case status (left columns) and death among SJS/TEN cases (right columns), using all reports in FAERS from 2013–2023 (n = 13,986,839). OR = odds ratio, CI = confidence interval, Dev = development, mort = mortality, ref = reference level.

To evaluate risk factors for the development of SJS/TEN and mortality in SJS/TEN patients, we conducted two multivariable logistic regressions; the first included all reports, modeling SJS/TEN occurrence as the outcome., and the second was restricted to reports with documented SJS/TEN, modeling death as the outcome. Both models included age, sex, number of concomitant drugs, cancer diagnosis, ICI exposure, and exposure to strong or weak culprit drugs as covariates (Figure 3). In the development model, ICI exposure was associated with a markedly increased risk of SJS/TEN (OR = 6.69 [6.19–7.23]), as was strong culprit (OR = 14.24 [13.71–14.79]) and weak culprit exposure (OR = 2.60 [2.46–2.74]). Risk declined with increasing age, with ORs ranging from 0.64 for those aged 21–40, to 0.40 for age 41–60, compared to age 0–20. Interestingly, cancer diagnosis was associated with lower risk (OR = 0.64 [0.61–0.67]). To determine whether drug exposures moderate this effect, we removed culprit and ICI exposure variables from the model, which increased the OR of cancer diagnosis to 1.02 [0.98–1.06]; this suggests some of the risk is mediated by drug exposure (eFigure 4).

In the mortality model, ICI exposure was independently associated with higher odds of death among SJS/TEN cases (OR = 1.19 [1.01–1.40]). Risk of death increased sharply with age, rising from an OR of 1.37 in patients aged 21–40 to 5.86 in those over 80 compared to patients aged 0–20. Cancer diagnosis was associated with increased mortality (OR = 1.77 [1.59–1.97]), in contrast to its inverse association with SJS/TEN development. Both strong and weak culprit exposure predicted increased mortality (OR = 1.09 [1.00–1.19] and 1.42 [1.26–1.60], respectively).

**Figure 4.**
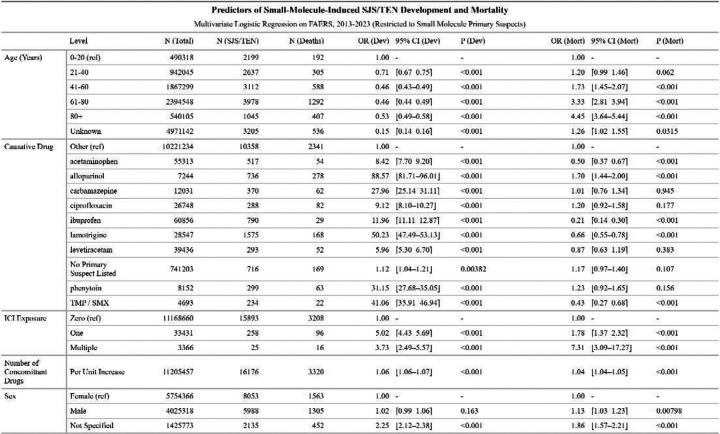
Predictors of Small-Molecule-Induced SJS/TEN Development and Mortality. Multivariable logistic regression was performed on all FAERS reports in which the primary suspect drug was a small molecule (n = 11,205,457), assessing factors associated with SJS/TEN (left columns) and death among SJS/TEN cases (right columns). Top 10 primary suspect drugs by frequency were modeled individually, with all others grouped as “Other”. OR = odds ratio, CI = confidence interval, Dev = development, mort = mortality, ref = reference level.

To further investigate predictors of SJS/TEN development and associated mortality, we conducted two additional multivariable logistic regressions, one with SJS/TEN as the outcome, limited to reports where a small molecule was listed as the primary suspect, and the other with death as the outcome, restricted to SJS/TEN cases with a small molecule as the primary suspect (Figure 4). Unlike the broader models, which evaluated exposure to pre-selected culprits, these models included the ten most common causative small molecules as covariates, with all other drugs grouped as the reference category. Additionally, ICI exposure was modeled with greater granularity using a three-level factor: no ICI exposure, exposure to a single ICI, and exposure to multiple ICIs. Other covariates included age, sex, and number of concomitant drugs.

In small-molecule-attributed cases, age was associated with lower SJS/TEN risk, with ages 41–60 (OR = 0.46 [0.43–0.49]) and 61–80 (OR = 0.46 [0.44–0.49]) having lower odds compared to age <20. Drugs *a priori* selected as strong culprits demonstrated strikingly increased SJS/TEN risk when listed as primary suspects, including allopurinol (OR = 88.57 [81.71–96.01]), lamotrigine (OR = 50.23 [47.49–53.13]), TMP-SMX (OR = 41.06 [35.91–46.94]), and phenytoin (OR = 31.15 [27.68–35.50]). Exposure to one (OR = 5.02 [4.43–5.69]) or multiple (OR = 3.73 [2.49–5.57]) ICI is independently associated with developing SJS/TEN. Mortality increases with age, with patients over 80 having an OR of 4.45 [3.64–5.44] relative to the youngest group. Some agents (e.g., acetaminophen, ibuprofen, TMP/SMX, lamotrigine) were associated with lower mortality despite elevated development risk. Allopurinol uniquely is associated with increased mortality (OR = 1.70 [1.44–2.00]). Importantly, ICI co-exposure is independently associated with mortality, with multiple ICI exposure (OR = 7.31 [3.09–17.27]) most strongly associated.

To assess confounding by indication, we performed additional regressions for SJS/TEN risk and mortality (as above) but restricted to cancer patients for whom a small molecule was the primary suspect of their adverse event (eFigure 5). Patterns were consistent with the general small-molecule-suspect population, including decreasing risk and increasing mortality with age. The most causative drugs, however, are quite different. While allopurinol (OR = 69.09 [52.70–90.56]) is strongly associated with the development of SJS/TEN in this population, there are also strong signals from antineoplastic agents of various classes, including the anti-hormonal apalutamide (OR = 13.24 [10.04–17.47], the alkylating agents bendamustine (OR = 7.90 [5.97–10.45]) and carboplatin (OR = 1.72 [1.40–2.13]), the antimetabolites methotrexate (OR = 4.45 [3.48–5.71]), and the kinase inhibitor vemurafenib (OR = 9.34 [7.30–11.94]). Like in the general population, allopurinol is associated with increased mortality risk (OR = 3.04 [1.79–5.18]), along with a handful of other agents. ICI exposure continued to strongly predict both SJS/TEN development (OR = 5.49 [4.77–6.32] for single-agent; OR = 4.78 [3.19–7.16] for multiple ICIs) and mortality (OR = 1.57 [1.16–2.13] and 4.19 [1.77–9.90], respectively).

We additionally conducted a regression for SJS/TEN risk on the population of patients for which ICI is the primary suspect, using age, sex, number of concomitant drugs, and co-exposure to strong and weak culprits as covariates (eFigure 6). Exposure to a strong culprit (allopurinol, lamotrigine, TMP-SMX, phenytoin, or carbamazepine) increased the odds of developing SJS/TEN (OR = 3.00 [2.10–4.29]). Among causative agents, pembrolizumab was most implicated and had a significantly elevated adjusted odds ratio (OR = 1.96 [1.50–2.57]) compared to the reference atezolizumab. We’ve also conducted another regression with the same variables, but grouping ICIs by class - CTLA-4, PD-1, PD-L1, and multiple classes with one marked as primary suspect (eFigure 7). Patients with multiple ICI had an increased odds ratio versus CTLA-4 (OR = 3.62 [2.05–6.40]). Exposure to a strong culprit is positively associated with developing SJS/TEN in this analysis as well (OR = 2.95 [2.06–4.21]).

### The Effect of ICI on Drug Latency in SJS/TEN

**Figure 5.**
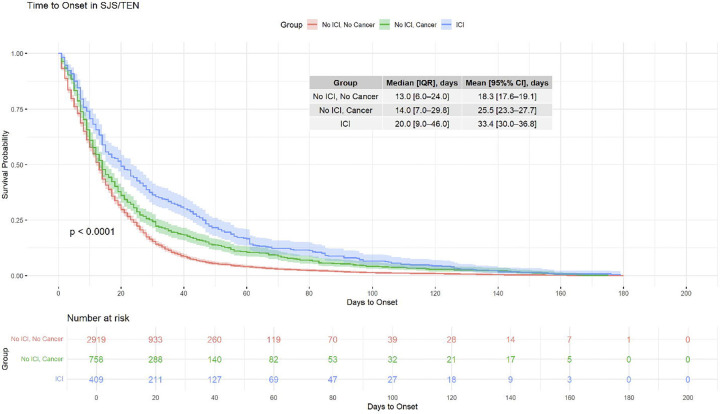

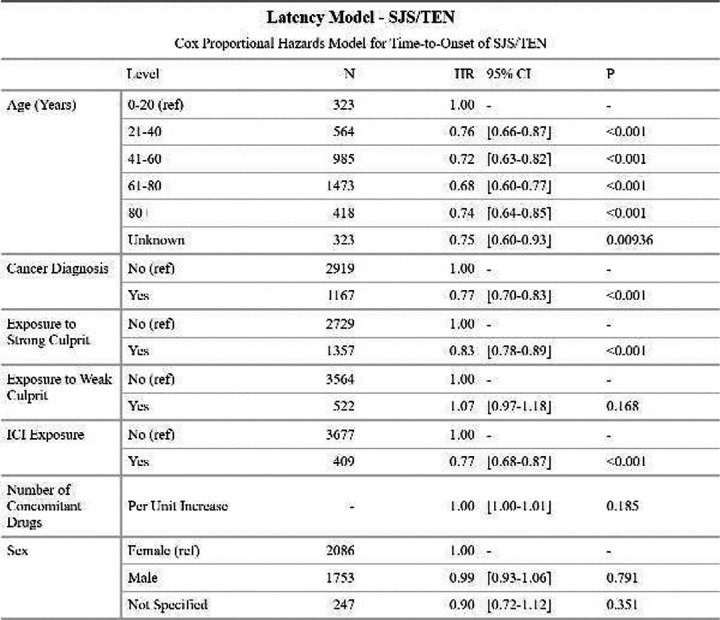
Latency in SJS/TEN by Clinical Exposure. (A) Kaplan-Meier curves on all SJS/TEN cases in FAERS with TTE information (n = 4,086) illustrate time to onset of SJS/TEN stratified by ICI exposure and cancer status. Note that all ICI-exposed patients also have a cancer diagnosis. Patients exposed to ICIs (blue) had longer latency compared to those with cancer but no ICI exposure (green) and those with neither cancer nor ICI exposure (red). P-value by log-rank test. (B) A multivariable Cox proportional hazards model including age, sex, number of concomitant drugs, cancer diagnosis, strong culprit exposure, and weak culprit exposure, identified ICI exposure as a predictor of delayed SJS/TEN onset. IQR = interquartile range, CI = confidence interval, HR = hazard ratio, ref = reference level.

We next analyzed TTE for all SJS/TEN cases with available latency information (n = 4,086, Figure 5). A Kaplan-Meier analysis shows that median latency is prolonged in patients exposed to ICI, compared to cancer patients not exposed to ICI and non-cancer patients (median, 20 vs 14 vs 13 days; log-rank P < .0001). In a Cox proportional hazards model, ICI exposure (HR = 0.77 [0.68–0.87], P < 0.001) and cancer diagnosis (HR = 0.77 [0.70–0.83], P < 0.001) was independently associated with delayed onset when controlling for sex, age, number of concomitant drugs, and culprit exposure. Older patients showed modestly reduced hazard of earlier onset, with HR 0.68 [0.60–0.77] for patients aged 61–80, and HR 0.74 [0.64–0.85] for those >80 (both P < 0.001). This effect is even more pronounced in a latency analysis separating by the class of causative agent, ICI vs non-ICI biologic vs small-molecule (eFigure 8). Cases with ICI as a primary suspect of SJS/TEN had a median of 23.5 days latency, which is substantially increased compared to non-ICI biologics (15.0 days) and small molecule drugs (13.0 days, log-rank P < 0.0001). The corresponding Cox model including age, cancer diagnosis, causative drug class, number of concomitant drugs, and sex recapitulated this difference, with ICI having substantial delay (HR = 0.74 [0.65–0.86], P < 0.001) compared to small molecule drugs.

In a separate TTE analysis restricted to small-molecule-induced cases (n = 3,654, eFigure 9), ICI exposure was associated with delayed onset (median 15 vs 13 days, log-rank p < 0.0001). Latency varied by causative agent, with ibuprofen (HR = 2.60 [2.02–3.34]), TMP-SMX (HR = 2.38 [1.83–3.08]), and ciprofloxacin (HR = 2.20 [1.68–2.87]) linked to faster onset, and allopurinol (HR = 0.79 [0.66–0.95]), lamotrigine (HR = 0.77 [0.69–0.87]), lenalidomide (HR = 0.71 [0.56–0.90]), and phenytoin (HR = 0.69 [0.55–0.87]) associated with delayed onset. A separate analysis of small-molecule-induced SJS/TEN in cancer patients (n = 792, eFigure 10) recapitulates many of these findings, including delayed onset with age, but ICI exposure is significantly associated with onset (log-rank P = 0.033), though with decreased effect size (median 15 vs 14 days); when controlling for other variables in the corresponding Cox model, this effect became not significant (0.90 [0.70–1.15, P = 0.395). Capecitabine (HR = 4.52 [2.66–7.69]), regorafenib (HR = 2.29 [1.35–3.87]), and vemurafenib (HR = 1.54 [1.07–2.21]) are associated with faster onset. Lenvatinib (HR = 0.65 [0.42–1.03]) and lenalidomide (HR = 0.88 [0.68–1.13]) showed non-significant trends toward delayed onset.

## Discussion

We have comprehensively analyzed the effects of ICI exposure on SJS/TEN using a decade of pharmacovigilance data. Through multivariate regression, we find that ICI exposure is significantly associated with increased risk, delayed onset, and higher mortality in reported SJS/TEN. We also find that, in cases with ICI as primary suspect, exposure to known small-molecule culprits of SJS/TEN independently increases risk. This supports a two-hit mechanism in which ICI creates a permissive environment for the development of immune-mediated drug reactions.

Disproportionality analysis and multivariate models revealed that ICI exposure increased SJS/TEN risk and mortality after adjusting for covariates. Strong signals were observed for combinations involving TMP-SMX, lamotrigine, and allopurinol, which are well-documented in the literature.^5^ The co-prescription of ICIs and sulfasalazine or azithromycin also showed striking risk elevations, though these were based on small counts and should be interpreted cautiously. Previous *in vitro* studies have found that checkpoint inhibition primes sulfamethoxazole- and sulfasalazine-specific T cells, corroborating these findings.^16,17^ Similarly, ICI has been reported to potentiate vemurafenib-induced drug reaction with eosinophilia and systemic symptoms (DRESS), and a series of patients with lichenoid mucocutaneous reactions to ICI had 80% co-exposure to known causes of lichenoid drug eruptions.^18–21^

Multivariate models on the broader FAERS population revealed ICI exposure independently increased SJS/TEN risk, even when known culprit drugs and cancer diagnosis were controlled for. Interestingly, cancer diagnosis was associated with reduced SJS/TEN risk in several models, including those focusing on patients exposed to TMP-SMX or allopurinol. However, when ICI and culprit exposure were removed, this association disappeared. Previous literature has suggested that cancer patients have increased risk of SJS/TEN.^22,23^ The association may be specific for certain cancers or correlated with cancer severity, and in particular has been previously associated with hematologic malignancies.^22,24^ These findings imply a nuanced relationship between malignancy and SJS/TEN, shaped by therapeutic context, pharmacologic triggers (antineoplastics, allopurinol for tumor lysis syndrome, or TMP/SMX for prophylaxis), and underlying disease, that requires thoroughly phenotyped data for further analysis.

TTE analyses added a complementary dimension. In the general population, ICI exposure was associated with substantially delayed onset, even after adjusting for age, sex, drug exposures, and cancer diagnosis (which was also associated with delayed TTE, and this effect is even more stark when comparing patients for whom ICI is coded as the primary culprit. Consistent with this finding, previous literature has shown that ICI-induced reactions coded as SJS/TEN have delayed onset.^14^ This effect was recapitulated when analyzing the effect of ICI exposure on TTE in small-molecule induced SJS-TEN, though it diminished when further subsetting to cancer patients, which is consistent with other TTE analyses showing that cancer diagnosis is associated with delayed onset. Additionally, several small-molecule drugs demonstrated latency consistent with prior studies, including rapid onset with agents like TMP-SMX and ciprofloxacin, and delayed reactions with allopurinol, lamotrigine, and phenytoin.^7^

Our study has certain limitations. First, FAERS is subject to underreporting, duplicate entries, and reporting biases, particularly related to newer or high-profile drug classes like ICIs.^25,26^ We attempted to mitigate these, but biases remain, and more advanced deduplication could improve these results.^27–29^ Also, FAERS lacks comprehensive histories, or laboratory confirmation of diagnosis; we thus cannot exclude misdiagnosis, incomplete drug or indication lists, and insufficient follow-up as a source of bias. In particular, ICI-induced SJS/TEN-like reactions can be clinically and pathologically distinct from small molecule SJS/TEN, and ICI-induced bullous or lichenoid reactions can imitate SJS/TEN; there is no distinction in FAERS between “true” SJS/TEN and SJS/TEN-like reactions, thus we assume they’re reported together for the sake of this analysis.^8,30–33^ Further studies should focus on well-phenotyped cohorts that can be distinguished clinically and histologically. Third, latency analyses rely on documentation of start dates, which can be inconsistent. Fourth, many ICI-culprit combinations had low counts, limiting generalizability, as there were no cases with ICI and carbamazepine or oxcarbazepine co-exposure. Studies of other databases, chart reviews, and prospective studies are needed to solidify the conclusions. Finally, we were unable to reliably code for cancer type and severity, so some findings may be partly mediated by the severity of the underlying condition or differences in the underlying biology of the neoplasm.

In aggregate, our findings highlight a pattern in which ICIs may act as both direct triggers of SJS/TEN, and facilitate reactions to small molecules, potentially by disrupting tolerance to drug antigens and lowering the threshold of T cell activation, leading to delayed-onset reactions resembling SJS/TEN.^16,17^ These results reinforce the importance of monitoring in patients receiving ICI, particularly when prescribing high-risk medications. They also suggest that SJS/TEN risk stratification in oncology should not be limited to individual drug profiles but should additionally incorporate the immunologic milieu created by ICI in concert with the tumor microenvironment.

## Supplementary Material

1 **eFigure 1. Demographic and Clinical Characteristics of ICI- vs Small-Molecule-Induced SJS/TEN.** Proportional distributions of (A) age, (B) sex, (C) reporting region, and (D) presence of strong culprits are shown for SJS/TEN cases attributed to immune checkpoint inhibitors (ICI) versus small molecules (SM). F = female, M = male, NS = not specified, PS = primary suspect.
**eFigure 2. Risk Factors for SJS/TEN Among Patients Exposed to Allopurinol.** Multivariable logistic regression was performed among 121,698 allopurinol-exposed cases reported to FAERS from 2013–2023 to identify demographic and clinical factors associated with SJS/TEN. OR = odds ratio, CI = confidence interval, ref = reference level.
**eFigure 3. Risk Factors for SJS/TEN Among Patients Exposed to TMP-SMX.** Multivariable logistic regression was performed among 90,282 TMP-SMX-exposed cases reported to FAERS from 2013–2023 to identify demographic and clinical factors associated with SJS/TEN. OR = odds ratio, CI = confidence interval, ref = reference level.
**eFigure 4. Predictors of SJS/TEN Development and Mortality, with Fewer Covariates.** Multivariable logistic regression was used to assess factors associated with SJS/TEN case status (left columns) and death among SJS/TEN cases (right columns), using all reports in FAERS from 2013–2023 (n = 13,986,839). OR = odds ratio, CI = confidence interval, Dev = development, mort = mortality, ref = reference level.
**eFigure 5. Predictors of Small-Molecule-Induced SJS/TEN Development and Mortality in Cancer Patients.** Multivariable logistic regression was performed on 1,940,961 reports in FAERS from 2013–2023, restricted to cases with a cancer diagnosis and a small-molecule primary suspect drug. Odds ratios are shown for both SJS/TEN development (left columns) and mortality among SJS/TEN cases (right columns). OR = odds ratio, CI = confidence interval, Dev = development, mort = mortality, ref = reference level.
**eFigure 6. Predictors of ICI-Induced SJS/TEN Development and Mortality, Stratified by Individual ICI.** Multivariable logistic regression was performed on 147,965 FAERS reports from 2013–2023, restricted to cases where an ICI was the primary suspect of their adverse reaction. Atezolizumab served as the reference drug. OR = odds ratio, CI = confidence interval, Dev = development, mort = mortality, ref = reference level.
**eFigure 7. Predictors of ICI-Induced SJS/TEN Development and Mortality, Grouped by ICI Class.** Multivariable logistic regression was performed on 147,965 FAERS reports from 2013–2023, restricted to cases where an ICI was the primary suspect of their adverse reaction. Primary suspects were categorized into CTLA-4, PD-L1, PD-1, LAG-3 (of which there were no cases), or multiple ICI classes. OR = odds ratio, CI = confidence interval, Dev = development, mort = mortality, ref = reference level.
**eFigure 8. Latency of SJS/TEN by Causative Drug Class.** (A) Kaplan-Meier curves comparing time-to-onset of SJS/TEN across three drug groups (n = 4,086): small molecules (red), non-ICI biologics (green), and ICIs (blue). ICIs were associated with significantly longer latency compared to the other groups (log-rank P < 0.0001). (B) Cox proportional hazards model adjusted for age, sex, cancer diagnosis, and number of concomitant drugs confirms that ICI and non-ICI biologics have significantly reduced hazard of early SJS/TEN onset compared to small molecules. IQR = interquartile range, CI = confidence interval, HR = hazard ratio, ref = reference level.
**eFigure 9. Latency of Small-Molecule-Induced SJS/TEN.** (A) Kaplan-Meier curves comparing time-to-onset across two groups with small-molecule-induced SJS/TEN (n = 3,654): ICI exposed (red) and non-ICI exposed (green). ICI exposure was associated with significantly longer latency (log-rank P < 0.0001). (B) Cox proportional hazards model adjusted for age, sex, causative drug, and number of concomitant drugs confirms that ICI exposure significantly reduces hazard of early SJS/TEN (HR = 0.67, [0.55–0.82]). IQR = interquartile range, CI = confidence interval, HR = hazard ratio, ref = reference level.
**eFigure 10. Latency of Small-Molecule-Induced SJS/TEN in Cancer Patients.** (A) Kaplan-Meier curves comparing time-to-onset across two groups with small-molecule-induced SJS/TEN and cancer (n = 792): ICI exposed (red) and non-ICI exposed (green). ICI exposure was associated with significantly longer latency (log-rank P = 0.033). (B) Cox proportional hazards model adjusted for age, sex, causative drug, number of concomitant drugs, and ICI exposure. IQR = interquartile range, CI = confidence interval, HR = hazard ratio, ref = reference level.
**eFigure 11. Generalized Variance Inflation Factor (GVIF) Heatmap Across Regression Models.** GVIF values are shown for each predictor across multiple regression models assessing SCAR development and mortality. While some models approach a GVIF of 2 for age and sex, all values remain below the conventional multicollinearity concern threshold of 5, indicating acceptable collinearity. Color intensity reflects the degree of GVIF inflation, with darker shades indicating higher values.

## Data Availability

Methods and data are available at https://github.com/capuhcheeno/SCARs_ICI-Manuscript-Scripts/tree/main/ICI%20and%20Culprit%20Analysis

## Key Points

### Question:

Do immune checkpoint inhibitors (ICIs) facilitate Stevens-Johnson Syndrome and Toxic Epidermal Necrolysis (SJS/TEN), and do they synergize with known culprit drugs or impact disease latency?

### Findings:

In this cross-sectional study of 17,495 SJS/TEN cases from 13,986,839 deduplicated FAERS reports (2013–2023), ICI exposure, independent of exposure to known culprits, increased the risk of developing SJS/TEN and amplified risk when co-administered with known culprits such as allopurinol and trimethoprim-sulfamethoxazole. ICI-exposed patients also showed significantly delayed onset compared to non-exposed cases.

### Meaning:

These findings suggest that ICIs directly contribute to SJS/TEN, likely both as a direct cause and through mechanistic synergy with antigenic small molecule drugs. These findings underscore the need for heightened vigilance when combining ICIs with high-risk medications and for recognizing atypical, delayed presentations.
