# Immune Checkpoint Inhibitors as Independent and Synergistic Drivers of SJS/TEN: An Analysis of FAERS

**DOI:** 10.1101/2025.06.21.25330030

**Published:** 2025-08-08

**Authors:** Eric Milan Mukherjee, Dodie Park, Amir Asiaee, Matthew S. Krantz, Cosby A. Stone, Michelle Martin-Pozo, Elizabeth Phillips

**Affiliations:** 1 Department of Dermatology, Vanderbilt University Medical Center, Nashville, Tennessee, USA; 2 Center for Drug Safety and Immunology, Vanderbilt University Medical Center, Nashville, Tennessee, USA; 3 Department of Biostatistics, Vanderbilt University Medical Center, Nashville, Tennessee, USA; 4 Department of Medicine, Division of Infectious Diseases, Vanderbilt University Medical Center, Nashville, Tennessee, USA; 5 Institute for Immunology and Infectious Diseases, Murdoch University, Perth, Australia

**Keywords:** Severe Cutaneous Adverse Reactions, Stevens-Johnson Syndrome, Toxic Epidermal Necrolysis, Checkpoint Inhibitors, Pharmacovigilance, FAERS

## Introduction

Immune checkpoint inhibitors (ICIs) are paradigm-shifting cancer treatments that are increasingly associated with Stevens-Johnson Syndrome, Toxic Epidermal Necrolysis (SJS/TEN) and other life-threatening cutaneous reactions. Differentiating ICI-induced “true” SJS/TEN from SJS/TEN-like reactions is difficult, the latter of which may be distinct lichenoid or bullous reactions.^1–3^ In some cases, ICI-related-SJS/TEN-like reactions occur in association with HLA restricted drug culprits like allopurinol, suggesting a “two-hit” mechanism.^4,5^ With increasing ICI use, a clearer understanding of their role in SJS/TEN is critical.

## Methods

We analyzed 13,986,839 deduplicated FAERS reports (2013–2023), containing 17,495 SJS/TEN cases. We assessed the impact of ICI using logistic regressions adjusted for age, sex, cancer, polypharmacy, strong (lamotrigine, TMPSMX, phenytoin, allopurinol, carbamazepine) or weak (azithromycin, clarithromycin, erythromycin, ciprofloxacin, levofloxacin, moxifloxacin, and acyclovir) culprit exposure.

To assess latency patterns, we performed Cox proportional hazards analyses among SJS/TEN cases with documented latency. In Model 1, we used time-dependent Cox regressions with interval splitting to dynamically update exposure to PD-1, PD-L1, CTLA-4, or LAG-3 inhibitors. In Model 2, we compared latency between ICI-attributed and non-ICI-attributed cases, classifying primary suspect (PS) by ICI mechanism and using the same covariates.

## Results

**Figure 1.**
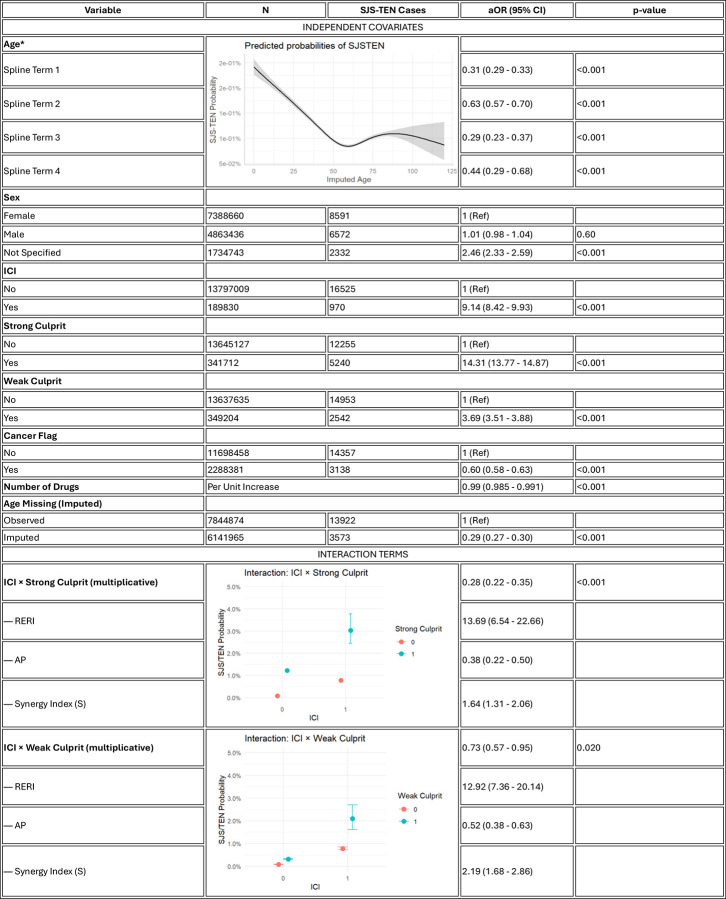
Multivariable logistic regression identifying independent and synergistic predictors of SJS/TEN. Adjusted odds ratios (aORs) with 95% confidence intervals are shown for independent covariates (age, sex, ICI use, strong and weak culprit drugs, cancer diagnosis, and polypharmacy) and for interaction terms between ICIs and culprit drug classes. Age was modeled using natural splines with 4 degrees of freedom. Additive interaction was quantified using the relative excess risk due to interaction (RERI), attributable proportion (AP), and synergy index (S). Notably, ICIs had a strong independent association with SJS/TEN (aOR 9.14) and exhibited substantial synergy with both strong and weak culprit drugs (RERI 13.69 and 12.92, respectively).

In a multivariable logistic regression (Figure 1), ICI exposure was strongly associated with increased risk of SJS/TEN (adjusted OR [aOR]: 9.14, 95% CI: 8.42–9.93, p < 0.001). Strong culprit drugs were the most potent independent predictors (aOR: 14.31, 13.77–14.87). Interestingly, cancer diagnosis was inversely associated with SJS/TEN risk (aOR: 0.60, 0.58–0.63). Interaction terms revealed additive synergy between ICI exposure and culprit drugs. The ICI × strong culprit interaction yielded an attributable proportion (AP) of 0.38, indicating that 38% of the risk in co-exposed patients is attributable to interaction. For ICI × weak culprits, the AP was even higher (0.52).

**Figure 2.**
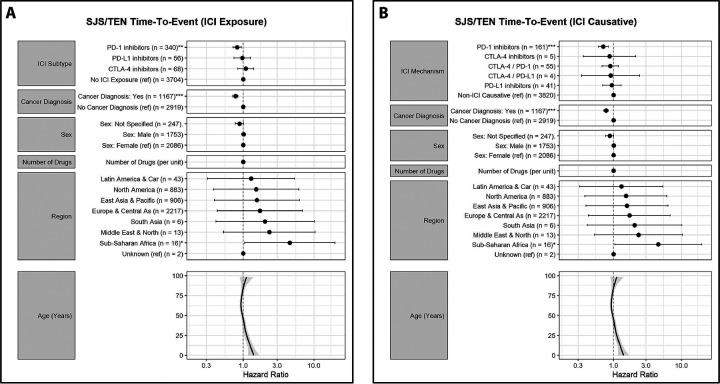
Latency Patterns of SJS/TEN in Relation to ICI Exposure and Mechanism of Causation. (A) Time-dependent Cox regression assessing the effect of immune checkpoint inhibitor (ICI) exposure on time to SJS/TEN onset. PD-1 inhibitors were significantly associated with delayed onset (HR 0.82, p < .01). Cancer diagnosis (HR 0.78, p < .001) and higher drug counts per case were also associated with longer latency. The bottom panel shows a spline-based effect of age, indicating modest delay in older patients. (B) Cox regression comparing SJS/TEN onset among cases where the primary suspect was an ICI versus a non-ICI drug. PD-1 inhibitors again showed significant latency prolongation (HR 0.71, p < .001), with similar trends seen for combination ICIs. Covariate effects mirrored Panel A, supporting robust associations between ICI mechanism and delayed SJS/TEN onset.

Patients with an anti-PD-1 as PS had TTE of 27 days, compared to 13 days for non-ICI, 15 days for PD-L1, and 20 days for CTLA-4/PD-1 combination. We further evaluated latency patterns using two Cox models. In the time-dependent exposure model/Model 1 (Figure 2A), PD-1 inhibitors were associated with delayed SJS/TEN onset (HR 0.82, *p* < .01). Patients with cancer diagnoses had later onset (HR 0.78, *p* < .001), as did those with greater polypharmacy (HR <1 per drug). In the causative-agent model/Model 2 (Figure 2B), PD-1 inhibitors again exhibited delayed onset compared to non-ICI causative drugs (HR 0.71, *p* < .001). The direction and magnitude of covariate effects were consistent between models.

## Discussion

Our findings confirm that ICIs are independently associated with increased risk of SJS/TEN and can synergize with high-risk small molecules to further amplify this risk. Both strong culprits (e.g., allopurinol, TMP-SMX) and weaker culprits (e.g., fluoroquinolones, macrolides) were significant predictors of SJS/TEN, but their effects were substantially magnified in the presence of ICI exposure, supporting a model of additive or supra-additive risk. Furthermore, latency analyses revealed that ICI-associated SJS/TEN presents later than non-ICI cases, with anti-PD-1 therapies showing an onset period nearly two-fold longer. These latency effects were consistent across both time-dependent and causative-agent Cox models.

Together, these results suggest a “two-hit” model, in which ICIs lower threshold for drug-specific T-cell activation. This model is supported by emerging mechanistic evidence on HLA-restricted, T-cell mediated hypersensitivity, showing that ICI can decrease threshold for T-cell activation.^6^ The delayed onset observed with ICI-associated SJS/TEN may further contribute to misattribution, increasing the risk of under-recognition or misdiagnosis in oncology settings. From a clinical perspective, our findings underscore the importance of careful co-prescribing in ICI-treated patients. These insights also reinforce the need for prospective studies and pharmacogenomic investigations to identify patients at highest risk and to develop guidelines for safer prescribing in cancer immunotherapy.

## Data Preparation

We analyzed FAERS reports from 2013 to 2023 using a deduplicated dataset constructed as previously described.^1^ We restricted analyses to the post-ICI era (2013 onward) and excluded duplicate reports, following standard pharmacovigilance practices.

Drugs coded as primary suspect (PS) were considered causative for the sake of this analysis. TMP-SMX exposure was comprehensively captured by merging reports listing trimethoprim, sulfamethoxazole, or pre-combined formulations. Exposure variables were constructed for immune checkpoint inhibitors (ICIs) when present in the drug list for a patient in any role. ICIs were grouped by mechanistic class (PD-1, PD-L1, CTLA-4, LAG-3), and additional variables included polypharmacy (number of unique drugs), sex, age, and cancer diagnosis (assigned using both indication keywords and manual curation of cancer-specific medications).

Age was missing in 43.9% of entries. To preserve modeling power while respecting variable distribution, we imputed missing ages using sequential hot deck imputation via the impute_shd() function from the simputation package, using the ten most age-correlated variables as donors. This approach preserves covariate relationships without assuming distributional form.

For time-to-event analyses, we calculated latency (TTE, in days) as the interval between drug initiation and SJS/TEN symptom onset, using FDA-submitted dates. We retained events with full month, day, and year information for both primary suspect drug and event, with plausible latency values between 1 and 180 days. In total, 4,086 SJS/TEN cases had usable TTE data and were included in survival analyses.

## Logistic Regression Analysis

To assess whether immune checkpoint inhibitor (ICI) exposure modifies the association between small-molecule drugs and risk of SJS/TEN, we conducted a multivariable logistic regression that included interaction terms between ICI exposure and two drug classes: strong culprits (e.g., allopurinol, TMP-SMX) and weak culprits (e.g., macrolides, fluoroquinolones). The final model was specified as:


\text{logit}\left(P(\text{SJS/TEN})\right) = \beta_0 + \beta_1 \cdot \text{ICI} + \beta_2 \cdot \text{StrongCulprit} + \beta_3 \cdot (\text{ICI} \times \text{StrongCulprit}) + \beta_4 \cdot \text{WeakCulprit} + \beta_5 \cdot (\text{ICI} \times \text{WeakCulprit}) + f(\text{age}) + \beta_6 \cdot \text{sex} + \beta_7 \cdot \text{cancer} + \beta_8 \cdot \text{NumDrugs} + \beta_9 \cdot \text{ageMissing}


Where:

- f(age) is modeled using a natural spline with four degrees of freedom to capture nonlinear age effects.
- ageMissing is a binary variable indicating imputed vs. observed age, included to account for missing age values (imputed using sequential hot-deck imputation).
- Additional covariates included sex, cancer diagnosis, and polypharmacy (number of drugs).

We estimated multiplicative interaction effects for both ICI × Strong Culprit and ICI × Weak Culprit using interaction terms in the model. To assess additive interaction, we used the interactionR package to compute^2^:

- RERI (Relative Excess Risk due to Interaction)
  \text{RERI} = RR_{11} - RR_{10} - RR_{01} + 1
  where RR_{ij} denotes risk ratios for combinations of ICI (i) and culprit exposure (j).
- AP (Attributable Proportion due to interaction)
  \text{AP} = \frac{\text{RERI}}{RR_{11}}
- S (Synergy Index)
  \text{S} = \frac{RR_{11} - 1}{(RR_{10} - 1) + (RR_{01} - 1)}

These metrics quantify the degree of risk amplification due to combined ICI and culprit drug exposure, beyond the sum of their individual effects. Confidence intervals for RERI, AP, and S were derived using delta method standard errors as implemented in interactionR.

## Time-to-Event Analyses

We performed two complementary Cox proportional hazards models to assess latency patterns in SJS/TEN cases, using documented time-to-onset (TTE) from drug initiation to symptom onset.

### Model 1: Time-Dependent ICI Exposure

To address immortal time bias and examine mechanistic differences across immune checkpoint inhibitor (ICI) classes, we implemented a time-dependent Cox regression using interval-split data. Each patient’s follow-up time was divided into risk intervals corresponding to exposure status for PD-1, PD-L1, CTLA-4, and LAG-3 inhibitors before the event date. For those cases with only month or year information, admin date was coded as the beginning of the month or the year (Jan 1), respectively. Time-dependent binary indicators were updated dynamically at the start of each interval to reflect ICI administration timing to avoid immortal time bias. The model was fit using the following structure:


\lambda(t) = \lambda_0(t) \exp\left(\beta_1 \cdot \text{PD-1}_t + \beta_2 \cdot \text{PD-L1}_t + \beta_3 \cdot \text{CTLA-4}_t + \beta_4 \cdot \text{LAG-3}_t + f(\text{age}) + \boldsymbol{\beta} \cdot X\right)


Where:

- \( \lambda(t) \): Hazard function at time \( t \)
- \( \lambda_0(t) \): Baseline hazard at time \( t \)
- \( \text{PD-1}_t, \text{PD-L1}_t, \text{CTLA-4}_t, \text{LAG-3}_t \): Time-dependent ICI exposure indicators
- \( f(\text{age}) \): Penalized spline function for age (4 degrees of freedom)
- \( \boldsymbol{\beta} \cdot X \): Linear predictor for covariates (e.g., sex, cancer status, region, number of drugs)

This approach allowed for estimation of hazard ratios specific to ICI subclasses and identification of delayed versus early-onset risk patterns associated with different immunotherapy agents.

### Model 2: ICI vs Non-ICI Culprit Comparison

To compare latency distributions between ICI-attributed and non-ICI-attributed SJS/TEN cases, we restricted analysis to reports with clearly identified primary suspect drugs. We classified each causative agent as an ICI or non-ICI and further subtyped ICIs by mechanism (PD-1, PD-L1, CTLA-4, or combinations thereof). A standard Cox proportional hazards model was then constructed using TTE as the outcome:


λ(t)=λ0(t)ex (β.Mechanism+f(age)+β.X)\lambda(t) = \lambda_0(t) \exp\left(\beta \cdot \text{Mechanism} + f(\text{age}) + \beta \cdot X\right)


As above, covariates included penalized splines for age, along with sex, cancer status, polypharmacy, and region. Mechanism of action (e.g., PD-1, PD-L1, CTLA-4, CTLA-4/PD-1) was treated as a categorical variable with “non-ICI” as the reference group. ICIs administered in combination were collapsed into a single composite variable using the earliest shared start date and unique mechanism combinations (e.g., “CTLA-4 / PD-1”).

## Visualization and Interpretation

For logistic regressions, marginal effect plots for age and interaction terms were generated using the ggpredict R package. For Cox models, hazard ratios with 95% CI were extracted from each model and visualized via forest plots. Age effects were displayed separately using spline-predicted hazard functions, plotted against binned age values with ribbons representing ±1.96 standard error. Spline terms captured nonlinear associations between age and latency. All models were implemented in R using the survival, pspline, and broom packages.

## Data Availability

Data and methods are available at https://github.com/capuhcheeno/SCARs_ICI-Manuscript-Scripts/tree/main/ICI%20and%20Culprit%20Analysis.
