# Supplementary material for: Immune Checkpoint Inhibitors as Independent and Synergistic Drivers of SJS/TEN: A FAERS-Based Analysis
Source: medRxiv. 2025 Jun 30:2025.06.21.25330030. Preprint. [Version 2] doi: 10.1101/2025.06.21.25330030 (PMC12236933; doi:10.1101/2025.06.21.25330030)
Supplement: Supplementary file 1 [file NIHPP2025.06.21.25330030V2-supplement-1.pdf]

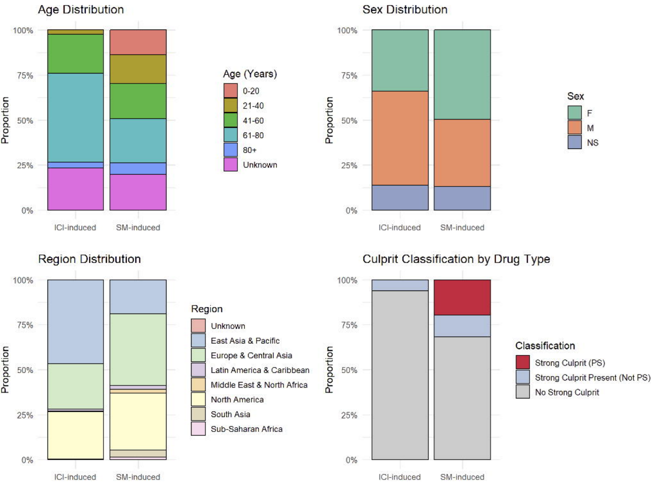
Age Distribution
100%
75%
50%
25%
0%
Proportion
ICI-induced
SM-induced
Age (Years)
0-20
21-40
41-60
61-80
80+
Unknown
Sex Distribution
100%
75%
50%
25%
0%
Proportion
ICI-induced
SM-induced
Sex
F
M
NS
Region Distribution
100%
75%
50%
25%
0%
Proportion
ICI-induced
SM-induced
Region
Unknown
East Asia & Pacific
Europe & Central Asia
Latin America & Caribbean
Middle East & North Africa
North America
South Asia
Sub-Saharan Africa
Culprit Classification by Drug Type
100%
75%
50%
25%
0%
Proportion
ICI-induced
SM-induced
Classification
Strong Culprit (PS)
Strong Culprit Present (Not PS)
No Strong Culprit

SJS/TEN in Patients Exposed to Allopurinol

Multivariable Logistic Regression, FAERS 2013-2023

		N (Total)	N (SJS/TEN)	Odds Ratio	95% CI	P-value
Age (Years)	0-20 (ref)	1217	18	1.00	-	-
	21-40	2844	78	1.45	[0.85 2.46]	0.171
	41-60	17242	229	0.87	[0.53–1.43]	0.595
	61-80	54590	660	0.89	[0.55 1.44]	0.624
	80+	15911	261	0.98	[0.60–1.60]	0.928
	Unknown	29894	196	0.19	[0.12 0.32]	<0.001
Sex	Female (ref)	39798	618	1.00	-	-
	Male	71900	600	0.49	[0.44 0.55]	<0.001
	Not Specified	10000	224	4.03	[3.27–4.96]	<0.001
Number of Concomittant Drugs	Per Unit Increase	121698	1442	0.87	[0.86 0.88]	<0.001
ICI Exposure	No (ref)	119360	1399	1.00	-	-
	Yes	2338	43	4.35	[3.12 6.06]	<0.001
Cancer Diagnosis	No (ref)	69898	1168	1.00	-	-
	Yes	51800	274	0.37	[0.32 0.43]	<0.001

SJS/TEN in Patients Exposed to TMP-SMX

Multivariable Logistic Regression, FAERS 2013-2023

		N (Total)	N (SJS/TEN)	Odds Ratio	95% CI	P-value
Age (Years)	0-20 (ref)	7969	91	1.00	-	-
	21-40	7976	141	1.18	[0.91–1.55]	0.215
	41-60	19503	255	1.04	[0.82–1.33]	0.738
	61-80	26872	236	0.77	[0.61–0.99]	0.0409
	80+	4386	94	1.72	[1.28–2.30]	<0.001
	Unknown	23576	143	0.37	[0.27–0.50]	<0.001
Sex	Female (ref)	41458	481	1.00	-	-
	Male	38653	374	0.87	[0.76–1.00]	0.0458
	Not Specified	10171	105	1.84	[1.41–2.41]	<0.001
Number of Concomittant Drugs	Per Unit Increase	90282	960	0.97	[0.97–0.98]	<0.001
ICI Exposure	No (ref)	88708	916	1.00	-	-
	Yes	1574	44	5.68	[4.05–7.95]	<0.001
Cancer Diagnosis	No (ref)	53536	745	1.00	-	-
	Yes	36746	215	0.39	[0.33–0.46]	<0.001

Predictors of SJS/TEN Development and Mortality in FAERS

Multivariate Logistic Regression on All FAERS Reports, 2013–2023

	Level	N (Total)	N (SJS/TEN)	N (Deaths)	OR (Dev)	95% CI (Dev)	P (Dev)	OR (Mort)	95% CI (Mort)	P (Mort)
Age (Years)	0-20 (ref)	580258	2226	196	1.00	-	-	1.00	-	-
	21-40	1199659	2696	312	0.62	[0.58–0.65]	<0.001	1.35	[1.11–1.63]	0.0021
	41-60	2460558	3389	651	0.35	[0.33–0.37]	<0.001	2.01	[1.69–2.39]	<0.001
	61-80	2984462	4522	1487	0.38	[0.36–0.40]	<0.001	3.86	[3.28–4.54]	<0.001
	80+	619937	1089	427	0.44	[0.41–0.48]	<0.001	5.72	[4.72–6.94]	<0.001
	Unknown	6141965	3573	621	0.12	[0.11–0.12]	<0.001	1.43	[1.18–1.75]	<0.001
Cancer Diagnosis	No (ref)	11698458	14357	2650	1.00	-	-	1.00	-	-
	Yes	2288381	3138	1044	1.02	[0.98–1.06]	0.444	1.81	[1.65–1.98]	<0.001
Number of Concomitant Drugs	Per Unit Increase	13986839	17495	3694	1.06	[1.06–1.07]	<0.001	1.04	[1.04–1.05]	<0.001
Sex	Female (ref)	7388660	8591	1685	1.00	-	-	1.00	-	-
	Male	4863436	6572	1501	1.14	[1.10–1.17]	<0.001	1.16	[1.07–1.26]	<0.001
	Not Specified	1734743	2332	508	2.69	[2.55–2.84]	<0.001	1.82	[1.55–2.13]	<0.001

Predictors of Small-Molecule-Induced SJS/TEN Development and Mortality (Cancer Patients)

Multivariate Logistic Regression on FAERS, 2013-2023 (Restricted to Small Molecule Primary Suspects With Cancer Diagnosis)

	Level	N (Total)	N (SJS/TEN)	N (Deaths)	OR (Dev)	95% CI (Dev)	P (Dev)	OR (Mort)	95% CI (Mort)	P (Mort)
Age (Years)	0-20 (ref)	49587	110	53	1.00	-	-	1.00	-	-
	21-40	63379	127	46	1.08	[0.83 1.41]	0.573	0.75	[0.43 1.30]	0.299
	41-60	308452	480	124	0.87	[0.70–1.08]	0.214	0.53	[0.32–0.86]	0.0106
	61-80	643573	912	317	0.81	[0.65 1.00]	0.0535	0.81	[0.51 1.29]	0.374
	80+	127518	98	41	0.50	[0.37–0.66]	<0.001	1.03	[0.56–1.91]	0.924
	Unknown	748452	458	121	0.34	[0.27 0.43]	<0.001	0.51	[0.30 0.87]	0.0127
Causative Drug	Other (ref)	1484454	1451	439	1.00	-	-	1.00	-	-
	allopurinol	731	62	36	69.09	[52.70 90.56]	<0.001	3.04	[1.79 5.18]	<0.001
	apalutamide	6662	55	31	13.24	[10.04–17.47]	<0.001	3.20	[1.79–5.73]	<0.001
	bendamustine	6387	52	18	7.90	[5.97 10.45]	<0.001	1.54	[0.85 2.78]	0.156
	capecitabine	43059	49	21	1.25	[0.94–1.67]	0.122	2.27	[1.26–4.09]	0.00622
	carboplatin	30051	102	28	1.72	[1.40 2.13]	<0.001	0.69	[0.42 1.12]	0.13
	lenalidomide	292087	159	31	0.68	[0.58–0.81]	<0.001	0.59	[0.39–0.90]	0.0133
	methotrexate	13717	76	45	4.45	[3.48 5.71]	<0.001	3.24	[1.88 5.60]	<0.001
	No Primary Suspect Listed	30752	69	32	1.82	[1.43–2.32]	<0.001	1.71	[1.03–2.83]	0.0382
	paclitaxel	25076	42	11	1.10	[0.81–1.50]	0.554	0.92	[0.45–1.86]	0.809
	vemurafenib	7985	68	10	9.34	[7.30 11.94]	<0.001	0.56	[0.28 1.11]	0.0989
ICI Exposure	Zero (ref)	1904164	1902	590	1.00	-	-	1.00	-	-
	One	33431	258	96	5.49	[4.77 6.32]	<0.001	1.57	[1.16 2.13]	0.00391
	Multiple	3366	25	16	4.78	[3.19–7.16]	<0.001	4.19	[1.77–9.90]	0.00111
Number of Concomittant Drugs	Per Unit Increase	1940961	2185	702	1.05	[1.04–1.05]	<0.001	1.05	[1.03–1.07]	<0.001
Sex	Female (ref)	858622	1038	305	1.00	-	-	1.00	-	-
	Male	852517	890	315	0.81	[0.74 0.88]	<0.001	1.23	[1.00 1.51]	0.0493
	Not Specified	229822	257	82	1.67	[1.42–1.96]	<0.001	1.55	[1.06–2.25]	0.0221

Predictors of ICI-Induced SJS/TEN Development and Mortality

Multivariate Logistic Regression on FAERS, 2013–2023 (Restricted to ICI Primary Suspects)

	Level	N (Total)	N (SJS/TEN)	N (Deaths)	OR (Dev)	95% CI (Dev)	P (Dev)	OR (Mort)	95% CI (Mort)	P (Mort)
Age (Years)	Unknown (ref)	44990	152	42	1.00	-	-	1.00	-	-
	0-20	500	0	-	0.00	[0.00-2.54e+143]	0.946	-	-	-
	21-40	4487	15	4	1.19	[0.69-2.06]	0.527	1.17	[0.32-4.25]	0.812
	41-60	28002	142	41	1.86	[1.43-2.41]	<0.001	1.32	[0.68-2.55]	0.416
	61-80	62594	322	127	1.84	[1.47-2.32]	<0.001	2.21	[1.22-4.00]	0.00912
	80+	7392	22	10	1.02	[0.64-1.62]	0.925	2.28	[0.81-6.41]	0.117
Causative Drug	atezolizumab (ref)	19033	74	18	1.00	-	-	1.00	-	-
	avelumab	1852	1	1	0.16	[0.02-1.12]	0.0649	7.70e+06	[0.00]	0.986
	cemiplimab	1560	11	6	1.83	[0.95-3.51]	0.0691	5.17	[1.34-19.94]	0.0171
	dostarlimab	397	1	0	0.67	[0.09-4.87]	0.694	0.00	[0.00]	0.988
	durvalumab	8730	23	3	0.75	[0.47-1.19]	0.221	0.51	[0.13-1.96]	0.326
	ipilimumab	14978	72	19	1.38	[0.99-1.92]	0.0545	1.48	[0.68-3.25]	0.322
	nivolumab	61655	225	68	1.05	[0.80-1.37]	0.721	1.79	[0.95-3.38]	0.0738
	pembrolizumab	39655	245	109	1.96	[1.50-2.57]	<0.001	3.41	[1.81-6.41]	<0.001
	retifanlimab	13	0	-	0.00	[0.00]	0.991	-	-	-
	tremelimumab	92	1	0	2.99	[0.41-21.83]	0.279	0.00	[0.00]	0.989
Exposure to Strong Culprit	No (ref)	145318	613	208	1.00	-	-	1.00	-	-
	Yes	2647	40	16	3.00	[2.10-4.29]	<0.001	1.04	[0.51-2.13]	0.913
Exposure to Weak Culprit	No (ref)	146580	642	219	1.00	-	-	1.00	-	-
	Yes	1385	11	5	1.13	[0.60-2.14]	0.697	1.08	[0.30-3.91]	0.909
Number of Concomittant Drugs	Per Unit Increase	147965	653	224	1.03	[1.01-1.05]	0.00378	1.08	[1.03-1.13]	0.00112
Sex	Female (ref)	50031	222	77	1.00	-	-	1.00	-	-
	Male	80171	340	118	0.95	[0.80-1.13]	0.59	1.01	[0.69-1.47]	0.974
	Not Specified	17763	91	29	1.95	[1.47-2.60]	<0.001	2.23	[1.06-4.67]	0.0336

Predictors of ICI-Induced SJS/TEN Development and Mortality

Multivariate Logistic Regression on FAERS, 2013 2023 (Restricted to ICI Primary Suspects, Grouped by Class)

	Level	N (Total)	N (SJS/TEN)	N (Deaths)	OR (Dev)	95% CI (Dev)	P (Dev)	OR (Mort)	95% CI (Mort)	P (Mort)
Age (Years)	Unknown (ref)	44990	152	42	1.00	-	-	1.00	-	-
	0-20	500	0	-	0.00	[0.00-6.67e+143]	0.946	-	-	-
	21-40	4487	15	4	1.12	[0.65-1.94]	0.68	1.22	[0.33-4.46]	0.763
	41-60	28002	142	41	1.75	[1.34-2.27]	<0.001	1.22	[0.63-2.35]	0.554
	61-80	62594	322	127	1.77	[1.40-2.22]	<0.001	2.10	[1.16-3.79]	0.0139
	80+	7392	22	10	1.02	[0.64-1.62]	0.936	2.43	[0.87-6.74]	0.0885
Causative ICI Class	CTLA-4 (ref)	8518	13	6	1.00	-	-	1.00	-	-
	PD-L1	28205	83	20	1.71	[0.95-3.07]	0.0747	0.23	[0.06-0.79]	0.0201
	PD-1	87822	411	158	3.01	[1.73-5.24]	<0.001	0.56	[0.18-1.80]	0.333
	Multiple	23420	146	40	3.62	[2.05-6.40]	<0.001	0.32	[0.10-1.05]	0.0609
Exposure to Strong Culprit	No (ref)	145318	613	208	1.00	-	-	1.00	-	-
	Yes	2647	40	16	2.95	[2.06-4.21]	<0.001	1.01	[0.50-2.02]	0.987
Exposure to Weak Culprit	No (ref)	146580	642	219	1.00	-	-	1.00	-	-
	Yes	1385	11	5	1.18	[0.63-2.22]	0.609	1.12	[0.31-4.06]	0.865
Number of Concomittant Drugs	Per Unit Increase	147965	653	224	1.02	[1.00-1.04]	0.0358	1.08	[1.03-1.13]	<0.001
Sex	Female (ref)	50031	222	77	1.00	-	-	1.00	-	-
	Male	80171	340	118	0.90	[0.76-1.07]	0.248	1.00	[0.69-1.45]	0.995
	Not Specified	17763	91	29	1.77	[1.33-2.35]	<0.001	1.73	[0.84-3.55]	0.138

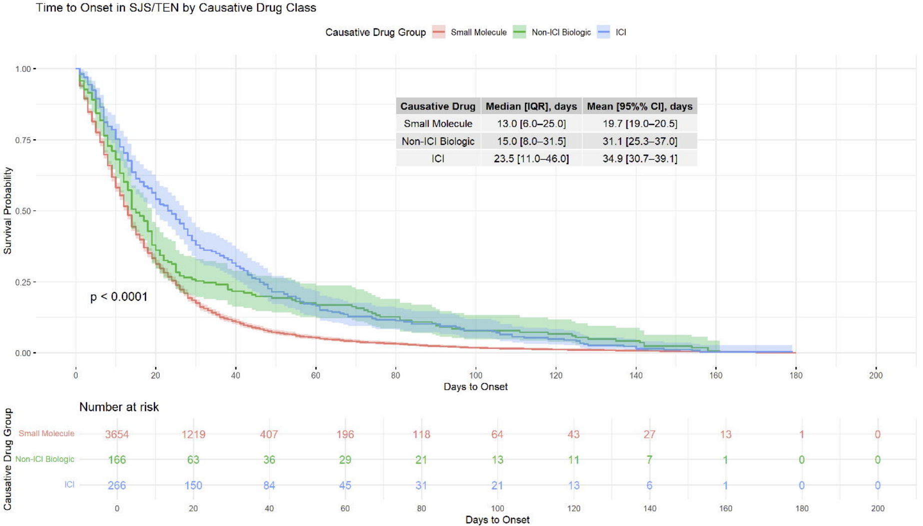
Time to Onset in SJS/TEN by Causative Drug Class
Causative Drug Group
Small Molecule
Non-ICI Biologic
ICI
Survival Probability
1.00
0.75
0.50
0.25
0.00
p < 0.0001
Causative Drug
Median [IQR], days
Mean [95%% CI], days
Small Molecule
13.0 [6.0–25.0]
19.7 [19.0–20.5]
Non-ICI Biologic
15.0 [8.0–31.5]
31.1 [25.3–37.0]
ICI
23.5 [11.0–46.0]
34.9 [30.7–39.1]
0 20 40 60 80 100 120 140 160 180 200
Days to Onset
Number at risk
Causative Drug Group
Small Molecule
3654 1219 407 196 118 64 43 27 13 1 0
Non-ICI Biologic
166 63 36 29 21 13 11 7 1 0 0
ICI
266 150 84 45 31 21 13 6 1 0 0
0 20 40 60 80 100 120 140 160 180 200
Days to Onset

Latency Model - SJS/TEN By Causative Drug Class

Cox Proportional Hazards Model for Time-to-Onset of SJS/TEN

	Level	N	HR	95% CI	P
Age (Years)	0-20 (ref)	323	1.00	-	-
	21-40	564	0.76	[0.67-0.88]	<0.001
	41-60	985	0.75	[0.66-0.85]	<0.001
	61-80	1473	0.70	[0.62-0.79]	<0.001
	80+	418	0.76	[0.65-0.88]	<0.001
	Unknown	323	0.78	[0.63-0.98]	0.0305
Cancer Diagnosis	No (ref)	2919	1.00	-	-
	Yes	1167	0.79	[0.73-0.85]	<0.001
Causative Drug Class	Small Molecule (ref)	3654	1.00	-	-
	Non-ICI Biologic	166	0.78	[0.67-0.92]	0.0023
	ICI	266	0.75	[0.65-0.86]	<0.001
Number of Concomitant Drugs	Per Unit Increase	-	1.00	[1.00-1.01]	0.23
Sex	Female (ref)	2086	1.00	-	-
	Male	1753	1.00	[0.94-1.06]	0.928
	Not Specified	247	0.89	[0.71-1.11]	0.291

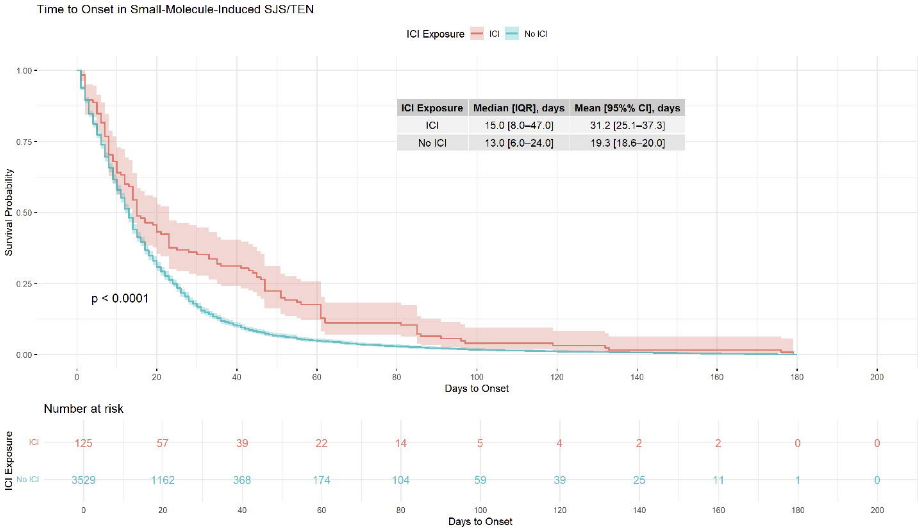
Time to Onset in Small-Molecule-Induced SJS/TEN
ICI Exposure
ICI
No ICI
ICI Exposure | Median [IQR], days | Mean [95%% CI], days
ICI | 15.0 [8.0–47.0] | 31.2 [25.1–37.3]
No ICI | 13.0 [6.0–24.0] | 19.3 [18.6–20.0]
p < 0.0001
Survival Probability
1.00
0.75
0.50
0.25
0.00
0
20
40
60
80
100
120
140
160
180
200
Days to Onset
Number at risk
ICI Exposure
ICI
125 57 39 22 14 5 4 2 2 0 0
No ICI
3529 1162 368 174 104 59 39 25 11 1 0
Days to Onset

Latency Model - Small-Molecule-Induced SJS/TEN

Cox Proportional Hazards Model for Time-to-Onset of SJS/TEN

	Level	N	HR	95% CI	P
Age (Years)	0-20 (ref)	320	1.00	-	-
	21-40	546	0.78	[0.68-0.90]	<0.001
	41-60	876	0.75	[0.65-0.85]	<0.001
	61-80	1220	0.65	[0.57-0.75]	<0.001
	80+	396	0.73	[0.62-0.85]	<0.001
	Unknown	296	0.79	[0.63-1.00]	0.053
Causative Drug	Other (ref)	2623	1.00	-	-
	allopurinol	129	0.79	[0.66-0.95]	0.0113
	carbamazepine	65	1.01	[0.79-1.30]	0.937
	carboplatin	47	0.95	[0.69-1.31]	0.76
	ciprofloxacin	55	2.20	[1.68-2.87]	<0.001
	ibuprofen	65	2.60	[2.02-3.34]	<0.001
	lamotrigine	394	0.77	[0.69-0.87]	<0.001
	lenalidomide	70	0.71	[0.56-0.90]	0.00442
	phenytoin	81	0.69	[0.55-0.87]	0.00133
	TMP / SMX	60	2.38	[1.83-3.08]	<0.001
	vancomycin	65	1.29	[1.01-1.65]	0.0453
ICI Exposure	No (ref)	3529	1.00	-	-
	Yes	125	0.67	[0.55-0.82]	<0.001
Number of Concomitant Drugs	Per Unit Increase	-	1.00	[1.00-1.01]	0.383
Sex	Female (ref)	1914	1.00	-	-
	Male	1511	0.94	[0.87-1.00]	0.0565
	Not Specified	229	0.84	[0.66-1.07]	0.158

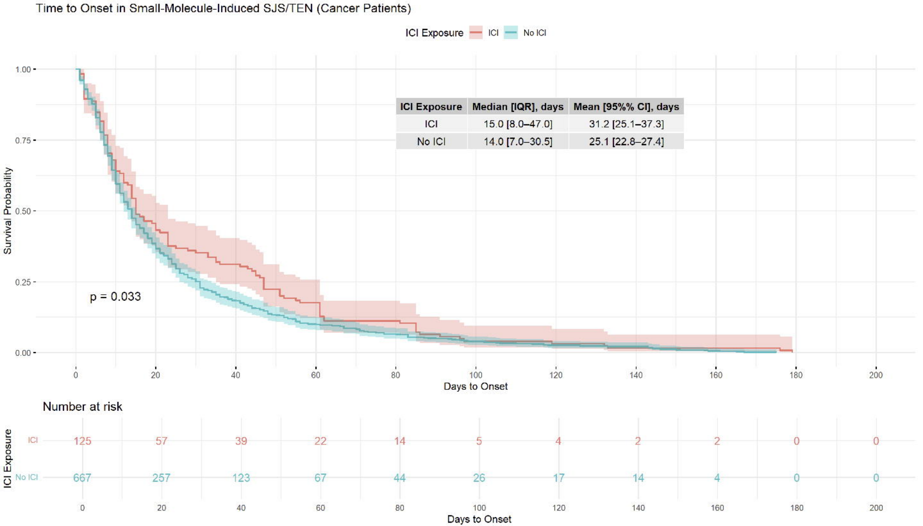
Time to Onset in Small-Molecule-Induced SJS/TEN (Cancer Patients)
ICI Exposure
ICI
No ICI
ICI Exposure | Median [IQR], days | Mean [95%% CI], days
ICI | 15.0 [8.0–47.0] | 31.2 [25.1–37.3]
No ICI | 14.0 [7.0–30.5] | 25.1 [22.8–27.4]
Survival Probability
1.00
0.75
0.50
0.25
0.00
p = 0.033
0
20
40
60
80
100
120
140
160
180
200
Days to Onset
Number at risk
ICI Exposure
ICI 125 57 39 22 14 5 4 2 2 0 0
No ICI 667 257 123 67 44 26 17 14 4 0 0
0
20
40
60
80
100
120
140
160
180
200
Days to Onset

Latency Model - Small-Molecule-Induced SJS/TEN in Cancer Patients

Cox Proportional Hazards Model for Time-to-Onset of SJS/TEN

	Level	N	HR	95% CI	P
Age (Years)	0-20 (ref)	23	1.00	-	-
	21-40	30	0.81	[0.47-1.41]	0.462
	41-60	212	0.71	[0.45-1.11]	0.133
	61-80	425	0.67	[0.43-1.04]	0.0776
	80+	51	0.65	[0.39-1.09]	0.102
	Unknown	51	0.75	[0.40-1.41]	0.369
Causative Drug	Other (ref)	517	1.00	-	-
	afatinib	14	1.25	[0.73-2.15]	0.412
	allopurinol	15	1.48	[0.88-2.49]	0.142
	apalutamide	21	0.86	[0.54-1.36]	0.515
	capecitabine	15	4.52	[2.66-7.69]	<0.001
	carboplatin	47	0.99	[0.71-1.40]	0.964
	lenalidomide	70	0.88	[0.68-1.13]	0.317
	lenvatinib	25	0.65	[0.42-1.03]	0.0657
	paclitaxel	19	1.33	[0.82-2.15]	0.248
	regorafenib	15	2.29	[1.35-3.87]	0.00204
	vemurafenib	34	1.54	[1.07-2.21]	0.0186
ICI Exposure	No (ref)	667	1.00	-	-
	Yes	125	0.90	[0.70-1.15]	0.395
Number of Concomitant Drugs	Per Unit Increase	-	1.01	[1.00-1.02]	0.0207
Sex	Female (ref)	405	1.00	-	-
	Male	352	0.90	[0.78-1.05]	0.175
	Not Specified	35	0.72	[0.41-1.27]	0.262

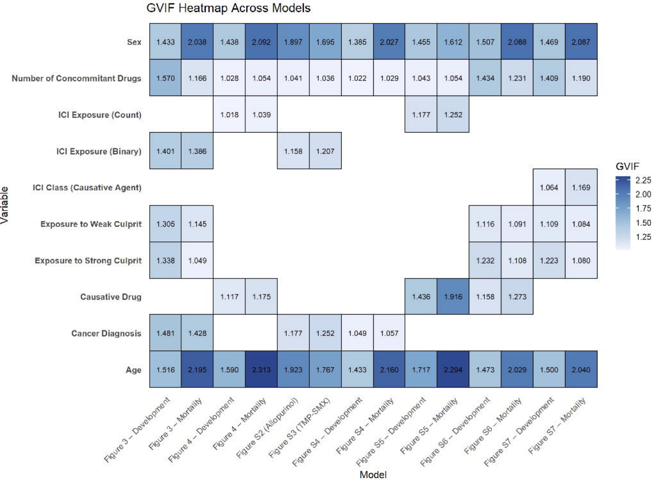

GVIF Heatmap Across Models
Sex
1.433 2.038 1.438 2.092 1.897 1.695 1.385 2.027 1.455 1.612 1.507 2.088 1.469 2.087
Number of Concommitant Drugs
1.570 1.166 1.028 1.054 1.041 1.036 1.022 1.029 1.043 1.054 1.434 1.231 1.409 1.190
ICI Exposure (Count)
1.018 1.039 1.177 1.252
ICI Exposure (Binary)
1.401 1.386 1.158 1.207
ICI Class (Causative Agent)
1.064 1.169
Exposure to Weak Culprit
1.305 1.145 1.116 1.091 1.109 1.084
Exposure to Strong Culprit
1.338 1.049 1.232 1.108 1.223 1.080
Causative Drug
1.117 1.175 1.436 1.916 1.158 1.273
Cancer Diagnosis
1.481 1.428 1.177 1.252 1.049 1.057
Age
1.516 2.195 1.590 2.313 1.923 1.767 1.433 2.160 1.717 2.294 1.473 2.029 1.500 2.040
Variable
Figure 3 – Development
Figure 3 – Mortality
Figure 4 – Development
Figure 4 – Mortality
Figure S2 (Allopurinol)
Figure S3 (TMP-SMX)
Figure S4 – Development
Figure S4 – Mortality
Figure S5 – Development
Figure S5 – Mortality
Figure S6 – Development
Figure S6 – Mortality
Figure S7 – Development
Figure S7 – Mortality
Model
GVIF
2.25
2.00
1.75
1.50
1.25
